# Regulation of data breach publication: the case of US healthcare and the HITECH act

**DOI:** 10.1007/s12197-022-09607-6

**Published:** 2022-11-17

**Authors:** Lorenz Bohn, Dirk Schiereck

**Affiliations:** Technische Universität Darmstadt, Darmstadt, Germany

**Keywords:** Data breach, Healthcare industry, HITECH act, Event study, G14, G18

## Abstract

Data breaches continue to plague most industries but especially the healthcare sector. While previous studies have aimed at assessing the financial implications arising from data breaches, none of them focused on the healthcare sector. This study of market reactions following data breaches fills that gap. Federal disclosure requirements of data breaches within the healthcare sector allow for our comprehensive study. Using multivariate regression analysis, our findings show the high specificity with which investors react to the announcement of data breaches.

## Introduction

The US healthcare sector is growing yearly, and Covid-19 even fosters this growth. The Centers for Medicare and Medicaid Services (2020) estimate that in 2019 the overall share of the U.S. gross domestic product related to health care spending was 17.7 percent. Similarly, the healthcare sector outperformed the S&P500 over the last ten years (S&P Dow Jones Indices 2021), underlining the growth potential and the size and heft of large healthcare companies and their impact on the healthcare sector in general. Compared to other industries, the advantages of leveraging data and the importance of protecting data became apparent early. The Health Insurance Portability and Accountability Act (HIPAA) of 1996 kickstarted the creation of national standards to protect sensitive patient health information and define permitted uses (Centers for Disease Control and Prevention 2018), while the Health Information Technology for Economic and Clinical Health Act (HITECH) of 2009 aimed at promoting and expanding the adoption of health information technology (U.S. Department of Health & Human Services 2017). But with the increasing adoption of electronic health and medical records, the criminal exploitation of patient data has increased correspondingly (Li et al. 2019). Due to the sensitive nature of the affected data, it is not surprising that the Ponemon Institute (2017) estimates the costs per breached record to be the highest in the healthcare sector. The susceptibility to data breaches and the enormous digital potential encapsulate the healthcare sector's current state. Previous studies made key contributions by exploring this subject from a variety of angles. Tanriverdi et al. (2020) show how different types of complexity within multihospital systems drive and mitigate the risks of data breaches while Angst et al. (2017) underline the importance of cohesive, deeply integrated IT security practices, by showing the persisting susceptibility of hospitals to IT risks if their efforts are merely symbolic. Building on these findings, Li et al. (2019) report that the deviation of security investments from the industry norms plays an important role in assessing the likelihood of security breaches.

This study aims to contribute to that area of research by analyzing the financial implications arising from data breaches, specifically stock market reactions to the announcement of data breaches. Previous studies of this type have typically assessed market reactions across multiple sectors or were heavily skewed towards IT firms. To the authors’ knowledge, there has not been a single comprehensive study focused on the healthcare sector. Given the importance of the US healthcare sector and the enforcement of data breach announcements in this sector, this analysis provides an important new viewpoint on IT and data in general in the healthcare sector. Due to federal disclosure requirements, the sample can be considered the most comprehensive sector-specific analysis of data breaches on firm value to date.

## Literature review and hypothesis

### Investor reactions over time

Investor reactions to data breaches have been the subject of multiple studies. In an analysis of 66 security breaches between 1996 and 2001, Cavusoglu et al. (2004) illustrate that a breached firm lost 2.1 percent of its market value within two days surrounding the event. Furthermore, using multivariate regression analysis, the findings indicate that investors reacted increasingly negative to more recent announcements. Based on a similar approach, Yayla and Hu (2011) divide their sample of 123 information-security-related events, into two sub-samples (1994 to 2000 and 2001 to 2006). Like Cavusoglu et al. (2004), the presented evidence indicates that e-commerce firms experience more negative stock price reactions. However, the findings also show that more recent events had a less significant impact, indicating that investors are becoming less sensitive to these types of announcements. Consequently, McShane and Nguyen (2020) hypothesize, that cyberattacks might have a time-variant effect on shareholder value. Their findings indicate that cumulative abnormal stock returns (CAR) follow a U-curve over time; showing increasingly negative reactions from 2007 until 2013, and increasingly less significant and even positive reactions thereafter.

To summarize, the existing evidence suggests changes in investor sensitivity regarding the announcement of data breaches. However, previous sample sizes also included disruptive events such as the dotcom-bubble or the financial crisis. Kannan et al. (2007) and Gordon et al. (2011) observe more negative reactions until December 2001, suggesting increased sensitivity following “9/11” while Modi et al. (2015) also report more negative reactions at the height of the financial crisis in 2009. Furthermore, Goel and Shawky (2014) analyze changes in investor reactions to security breaches before and after states in the U.S. adopted breach notification laws. Their findings underline a significant decline in negative returns once breach notifications became mandatory. Hence, an analysis of investor reactions in a period of prolonged recovery will provide a more up-to-date assessment of the current impact of data breach announcements on firm value:

- H1: There is a negative impact of data breaches on the stock price of affected companies, but this effect decreases over time.

### Sector dependency in market analysis

In 2017 the Ponemon Institute (2017) institute released a report, breaking down the data breach costs per stolen record. The estimated costs in the public sector were $71, in retail $154, services $223, and finance, the second-highest, $245 per breached record. The highest data breach costs per record occurred in the healthcare sector with $380. The expected long-term consequences of data breaches in the healthcare sector are estimated to be equally severe. The Ponemon Institute estimates the abnormal customer turnover following a data breach to rank second, only behind the finance sector. Even though the healthcare sector is often mentioned in discussions revolving around cybersecurity and digitalization, not to mention its economic importance in general, it is striking to realize that there exists hardly any insight with a strict focus on investor reactions following data breaches in the healthcare sector. There have been various studies based on multivariate analyses to assess industry sector-dependent stock market reactions (Amir et al. 2018; Andoh-Baidoo et al. 2010; Cavusoglu et al. 2004; Kannan et al. 2007; Malhotra and Kubowicz Malhotra 2010; McShane and Nguyen 2020; Michel et al. 2019; Pirounias et al. 2014; Yayla and Hu 2011). While some of them include healthcare firms, none of them assesses the impact of data breaches comprehensively in that sector. Gatzlaff and McCullough (2010) assess industry-sector specific market reactions following data breaches, including medical providers. However, the total number of cases involving medical providers (n = 6) is too low to allow for any inferences.

Considering the costliness of data breaches in healthcare, it is surprising to note that the extant literature has not been able to determine an industry-specific impact when it comes to healthcare. One possible explanation might be the variety of business models and business activity within healthcare, leading to the following hypothesis:

- H2: Within the healthcare sector, stock market reactions to data breaches differ depending on the specific business activity of the afflicted company.

### Confidentiality of data

Early studies have highlighted the importance of differentiating between the type of data affected by a breach. While Campbell et al. (2003) only find limited negative stock market reactions following public announcements of information security breaches between 1995 and 2000, they detect highly negative market reactions when the breach resulted in confidential data being accessed. Malhotra and Kubowicz Malhotra (2010) hypothesize that the type of information breached might lead to a different impact in terms of market value loss. The loss of financial information (i.e. credit card numbers, bank accounts, etc.) may result in direct financial losses for customers. However, these impacts can be mitigated by simple actions (e.g. cancellation of credit card), whereas personal information (e.g. social security numbers (SSN)) cannot be easily changed, possibly leading to higher costs in the long term. Ultimately, Bolster et al. (2010) as well as Malhotra and Kubowicz Malhotra (2010) do not find any significant impact related to the type of breached data. McShane and Nguyen (2020) show a significant negative impact in cases when credit card information or personal information (e.g. name, address, and email address) are accessed, but not in cases of SSNs.

As McShane and Nguyen (2020) summarize, the limited impact stemming from accessed SSN information is surprising. It is worth noting, however, that their analysis also accounts for industry sectors (retail, service, and financial). In contrast to Malhotra and Kubowicz Malhotra (2010) and Michel et al. (2019) they do not find significant, negative reactions in cases of data breaches in the financial sector. It is therefore plausible to imply some level of collinearity between financial firms and stolen credit card information. Hence, the impact of stolen SSN information should be reevaluated, especially when analyzing the impact of data breaches in the healthcare sector and the previously mentioned high costs per breached record:

- H3: Data breaches in the healthcare sector, in which SSNs were accessed, have a more negative impact on the share price of the affected company.

### Dependency on profitability and intangible assets

McShane and Nguyen (2020) clearly show the recent change in investor reaction to data breaches but stop short of providing a possible explanation for this trend. One might conclude that investors see little informational value in an event that nowadays occurs on such a regular basis. However, it seems reasonable to assume that investors have become more adept at assessing the consequences arising from data breaches. While the first instances of data breaches might be considered a seismic shift in the risk profile of an affected company, the risk of a data breach must be considered operational or almost a day-to-day event at this point. Consequently, data breaches are not comparable to other strategic corporate announcements, such as mergers, acquisitions, or spin-offs. Instead, data breaches could provide investors with additional insights into the process qualities of a company. In a study on privacy and security of healthcare data by the Ponemon Institute (2016), healthcare organizations listed employee negligence as the number one security concern. Similarly, Heath et al. (2021) show that an organization’s susceptibility to data breaches is highly dependent on sociotechnical variables. Investors might therefore conclude that a company susceptible to data breaches might also be prone to other types of risks resulting from employee mistakes or poor operational execution in general. Or, following Avery and Ranganathan (2016), breaches can be construed as opportunities to build organizational resilience and even result in long-term financial performance. While both propositions might seem contradictory at first, they point to an aspect that has been so far mostly neglected, management performance. This would imply that investor reaction to data breaches is heavily dependent on the general assessment of the management’s capabilities. If the management has performed well in the past investors might conclude that a data breach might be a blessing in disguise or at worst a non-event. A similar data breach afflicting a company with a history of poor management performance might reaffirm the negative prior:

- H4a: The negative impact of data breaches is smaller when the affected company’s management has performed well in the past.

When analyzing data breaches' short- and long-term consequences, Avery and Ranganathan (2016) introduce intangible assets as a new explanatory variable. They initially argue that a security breach is likely to affect the firm’s goodwill, brand reputation, and intellectual property. However, their findings show short- and long-term increases in intangible assets following a security breach which is interpreted as an indication of the limited impact of data breaches. This assessment might not be applicable to the healthcare sector. Intangible assets reflect a company’s past investments to build, maintain and/or expand its competitive advantage. By having a strong brand, a product offering protected by patents, or, like so many companies in the healthcare sector, hospital agreements, and customer relation assets, the company effectively shores up its competitive position. A higher share of intangible assets might therefore lead to more subdued investor reactions:

- H4b: The negative impact of data breaches is smaller when the affected company’s sales are secured by a high share of intangible assets.

## Research method

The financial impact of data breaches on companies is being assessed using an event study (MacKinlay 1997). This type of methodology is commonly utilized in accounting and finance studies, and increasingly in the IS domain (Dehning et al. 2003; Hinz et al. 2015). Campbell et al. (2003) and Cavusoglu et al. (2004) apply that methodology to study the effects of data theft on stock prices. Beyond the scope of data breaches, event studies are also used to examine the effects of IT investments (Dehning et al. 2003) and e-commerce announcements (Subramani and Walden 2001). Consequently, the event study methodology is a very capable and versatile tool to study the impact of news announcements on security prices (Hinz et al. 2015). In general, an event study investigates the effect of a given event, i.e. data breaches in the healthcare sector, on the respective company’s share price, specifically its’ share price returns. To assess the impact of an event, a given stock’s return that would have been observed in the absence of an event is being estimated by regressing the company’s stock market returns on the overall stock market index return. Using an ordinary least squares regression (OLS), a market model is then estimated over a period of 120 days before the defined event, thereby applying the most common estimation window (MacKinlay 1997) and following previous studies (Campbell et al. 2003; Hinz et al. 2015; Yayla and Hu 2011) that also use an estimation window of 120 days.

1 $${R}_{i,t} = {a}_{i} + {\beta }_{i} \times {R}_{m,t}+ {\varepsilon }_{i,t}$$

where ${R}_{i,t}$ is the return of a company *i*’s shares on day *t*. ${R}_{m,t}$ determines the stock market return on day *t*. ${a}_{i}$ and ${\beta }_{i}$ are the intercept and slope parameters for company *i*. Since all the companies, that are subject to this study, are traded on U.S.-stock exchanges, the S&P 500 is used as market index. By using the S&P 500 and not a more sector-specific index, any type of contagion or competition effects are minimized (Zafar et al. 2009). Next, to assess the capital market’s reaction, the abnormal stock return is calculated. The abnormal return (AR) is the difference between the expected return in the absence of an event, and the actual stock return that was realized at and around the event dates *t* = *-1, t* = *0 and t* = *1,2,3…*

2 $${AR}_{i,t} = {R}_{i,t} - ({ \widehat{a}}_{i}+ {\widehat{\beta }}_{i}{R}_{m,t})$$

The daily abnormal stock returns in the event period are estimated and cumulated. The cumulative abnormal return (CAR) represents the effect of a data breach on a company over an event window, whereby *t*_*1*_ and *t*_*2*_ represent the start and end date of the event window.

3 $${CAR}_{i}=\sum_{{t}_{1}}^{{t}_{2}}{AR}_{i,t}$$

To assess the statistical significance, a two-tailed *t*-test with the null hypothesis that the mean and median cumulated abnormal returns are equal to zero is employed. Next, building on this model, a multivariate OLS regression is implemented to determine factors that might influence the CAR, in our setting the number of affected individuals, a time variable, firm characteristics, financials as well as a dummy variable in case social security numbers were accessed.

4 $$\begin{array}{c}{CAR}_{i}={\beta }_{0}+{\beta }_{1}{Severity}_{i}{+ \beta }_{2}{Year}_{i}{ + \beta }_{3}Market{Cap}_{i}{ + \beta }_{4}Ro{E}_{i}\\ {+ \beta }_{5}Int{Assets}_{i}{ { + \beta }_{6}{DebtToCapital}_{i}+ \beta }_{7}{HospitalsClinics}_{i}{ + \beta }_{8}OtherHealthService{s}_{i}{ + \beta }_{9}{Retail}_{i}\\ {+ \beta }_{10}{ChemicalsAndMeasuring}_{i}{ + \beta }_{11}{Insurance}_{i}{ + \beta }_{12}SS{N}_{i}+ {\varepsilon }_{i}\end{array}$$

The *Severity* variable assesses the severity of a data breach based on the number of affected individuals. Due to the high numbers of smaller data breaches and the size of few but large data breaches the natural logarithm is applied to account for skewness. To construct the *Year* variable, a series of integer values for each year (e.g. 2012 = 1, 2013 = 2 etc.) is assigned in line with the extant literature (McShane and Nguyen 2020). A negative coefficient would indicate a negative reaction in more recent events, whereas a positive value would indicate less negative reaction from capital markets. Following Amir et al. (2018) and Yayla and Hu (2011) the model accounts for differences in market capitalization. Due to the level of skewness, the natural logarithm is again being used. The next variable, *RoE* is the return on equity based on the last full year report of a given company before the data breach. A similar approach is used for the variable *IntAssets*. Specifically, the intangible assets without goodwill are divided by the total sales of a given company. Multiple companies in the healthcare sector have intangible assets, ranging from hospital agreements and customer relations to patented technology. A positive significant coefficient would indicate that companies, whose sales are heavily dependent on intangible assets, are subject to less negative reactions following a data breach. In line with previous event studies, we deploy the variable *DebtToCapital* to account for firm leverage (Modi et al. 2015; McShane and Nguyen 2020) by dividing the total debt of a given company by its total capital. Coefficients 7 through 11 are dummy variables, i.e., 0 or 1, to enable a sub-industry sector-specific analysis. *HospitalsClinics* captures the following SIC Codes: 8050 Nursing Care Facilities, 8051 Skilled Nursing Facilities, 8060 Services Hospitals, 8062 General Medical and Surgical Hospitals. *HealthServices*: SIC 8011 Offices and Clinics of Doctors of Medicine, 8082 Home Health Care Services, 8090 Miscellaneous Health Services, 8093 Outpatient Facilities. *Retail*: SIC Codes starting with 51 (Wholesale trade-nondurable goods), 53 (General merchandise stores) and 59 (Miscellaneous Retail). *ChemicalsAndMeasuring*: SIC Codes starting with 28 (Chemicals and allied products) and 38 (Measuring, Analyzing, And Controlling Instruments). *Insurance*: SIC Codes starting with 63 (Insurance Carriers). The primary SIC (Standard Industry Classification) codes are based on the data provided by Refinitv Eikon. Lastly, the variable *SSN* is used to determine whether capital markets react more negatively to data breaches revolving around SSN. The research report from the U.S. Health and Human Services contains a web description when the investigation has been concluded. In those cases, the web description is searched for references of unauthorized access to social security numbers. In those cases, the dummy variable is set to 1, in all other instances, it is 0.

## Data

To assess the impact of data breaches on a firm’s capital market value requires data of related events. Before legislation enforcing the disclosure of these types of events, companies were not as forthcoming in informing the public of security events (Goel and Shawky 2014). Hence, earlier studies typically rely on research tools such as Lexis/Nexis or CNET (Cavusoglu et al. 2004; Yayla and Hu 2011), thereby most likely capturing only prominent events. As this study focuses on companies within the healthcare sector, the data provided by the U.S. Department of Health and Human Services (2021) is being used. Since February 22^nd^ 2010 any entity covered by the Health Insurance Portability and Accountability Act (HIPAA) has to notify the secretary of the U.S. Department of Health and Human Services and the affected individuals in the event of a security breach. In cases, where more than 500 individuals were affected the notification must occur without unreasonable delay. Based on these notifications the U.S. Department of Health and Human Services provides a regularly updated research report consisting of the covered entity, the covered entity type (e.g. healthcare provider, business associate, health plan etc.), the estimated number of affected individuals, the breach submission date, the type of breach and the location of the breached information (e.g. laptop, network server, desktop computer etc.). Due to the nationwide disclosure requirement, this report can therefore be considered the most comprehensive, sector-specific list of data breaches available without any bias toward larger events, media attention (Gordon et al. 2011; Michel et al. 2019) or material negative events (The SEC requires firms to disclose material negative events to investors. Firms beyond the healthcare sector are not required to disclose events with immaterial effects if they are compliant with state and federal disclosure requirements) (Amir et al. 2018).

The initial sample consists of 3,732 individual cases of data breaches, of which 295 involve publicly listed companies. The initial sample covers notifications submitted between October 21^st^ 2009 (before the effective date of the HITECH Act) and January 28^th^ 2021. Stock market and accounting information, such as the daily closing share prices, balance sheet data, stock market indices as well as general company information is retrieved from Refinitv Eikon. This data does not extend beyond October 1^st^ 2012, resulting in the exclusion of any event prior to that date and any event for which the calculation of the estimation window was not possible. The scope of the breach notification rule extends beyond the healthcare sector. For instance, companies primarily active in different business sectors may have to notify the secretary, if information of their employees’ health plan has been breached. Similarly, listed companies not traded on U.S. exchanges and companies, whose primary business activity is outside the healthcare sector are also excluded, bringing down the sample to 266. Further adjustments are needed in cases of one parent company submitting multiple notifications for individual subsidiaries resulting in a final sample size of 185 data breaches covering 43 firms in total. Tables 1 and 2 summarize the sample and the employed variables.

**Table 1:** Characteristics of sample firms

Industry sector	Events in sample	Percentage of sample	Avg. Market capitalization ($ millions)
Chemicals and Allied Products	6	3.2%	12,364.50
Measuring, Analyzing, and Controlling Instruments	5	2.7%	9,981.81
Wholesale Trade-Nondurable Goods	4	2.2%	23,130.12
General Merchandise Stores	10	5.4%	266,011.32
Miscellaneous Retail	28	15.1%	61,288.99
Insurance Carriers	94	50.8%	48,573.52
Business Services	2	1.1%	551.56
Health Services	34	18.4%	7,342.39
Engineering, Accounting, Research and Management	2	1.1%	4,062.02
Total events	185	100%	50,905.95

**Table 2:** Descriptive statistics

	Mean	SD	min	Q1	Q2	Q3	Max
Severity	8.58	2.05	6.22	7.19	8.18	9.27	18.18
Year	4.78	2.37	1.00	3.00	5.00	7.00	9.00
MarketCap	9.60	1.88	5.50	8.12	10.08	10.94	12.73
RoE	7.85	35.71	–445.14	5.48	11.14	17.37	51.86
IntAssets	9.62	17.99	0.00	0.96	4.96	9.79	100.00
DebtToCaptial	45.85	30.98	0.00	32.86	40.77	48.46	183.24
SSN	0.18	0.39	0.00	0.00	0.00	0.00	1.00

## Results

Table 3 provides an overview of the abnormal returns. In the first event period [-1, + 1] the mean cumulative abnormal return (CARCAPM) is + 0.20% and fairly close to the abnormal return [0,0] (+ 0.12%). While the third window [-1, + 3] is slightly higher at + 0.41%, whereas the fourth window [-3, + 3] is only slightly negative. While these results might seem counterintuitive, given the fact that data breaches should be considered negative events, they are in line with more recent findings of similar studies which similarly observed positive CARs in more recent time periods (Goel and Shawky 2014; McShane and Nguyen 2020). Furthermore, over half of the reported data breaches in this sample affected fewer than 4.000 individuals. Compared to other studies, especially those relying on information from Lexis/Nexis or CNET, these smaller cases would not have been part of a sample. Consequently, given the reach of big data breaches, it is fair to assume that investors would not typically base their investment decisions on minor data breaches affecting 501 or so individuals.

**Table 3:** Results of full sample statistical analysis using CAPM

Event window	Sample size	Median CAR	Mean CAR	*t* statistic	*p*-value
[-1, + 1]	185	–0.0011	0.0020	0.7688	0.4430
[0,0]	185	0.0007	0.0012	0.8480	0.3975
[-1, + 3]	185	0.0004	0.0041	1.2359	0.2181
[-3, + 3]	185	–0.0038	-0.0006	–0.1674	0.8672

Table 4 provides an overview of the multivariate analyses. Overall, the adjusted R^2^ indicates a high explanatory power compared with other recent studies of this type (Kamiya 2018; McShane and Nguyen 2020; Song et al. 2017). The variable *Severity* is negative and highly significant which indicates that investors consider the severity of a data breach. The variable *Year* is significant and positive which is in line with the findings of McShane and Nguyen (2020) that investors’ reactions to the announcement of a data breach is increasingly less negative or even positive. Overall, the results support hypothesis 1.

**Table 4:** Regression analysis of stock market reactions to data breaches

Variables	CARCAPM [–1 + 1]	CARCAPM [–1 + 1]	CARCAPM [–1 + 1]
Severity	–0.00316**	–0.00398***	–0.00396***
	(–2.435)	(–2.635)	(–2.607)
Year	0.00319*	0.00379**	0.00350**
	(1.861)	(2.200)	(2.154)
MarketCap	–0.00496**	–0.00521**	–0.00506**
	(–2.293)	(–2.465)	(–2.443)
RoE	0.000141***	0.000146***	0.000154***
	(3.600)	(2.619)	(2.648)
Int_Assets	2.50e-05	7.64e-05***	7.57e-05***
	(0.942)	(3.338)	(3.344)
DebtToCapital	3.74e-05	–8.11e-05	–8.25e-05
	(0.259)	(–0.574)	(–0.579)
HospitalsClinics		–0.000962	–0.00236
		(–0.0411)	(–0.0994)
OtherHealthServices		–0.0364	–0.0374
		(–1.238)	(–1.267)
Retail		–0.0178	–0.0216
		(–0.792)	(–0.920)
ChemicalsAndMeasuring		–0.0543*	–0.0579**
		(–1.955)	(–2.012)
Insurance		–0.0181	–0.0206
		(–0.777)	(–0.863)
SSN			–0.00813
			(–1.483)
Constant	0.0582**	0.0874**	0.0912**
	(2.539)	(2.177)	(2.200)
Observations	185	185	185
R–squared	0.101	0.187	0.193
Cluster	Robust	Robust	Robust
Adjusted R–squared	0.0702	0.135	0.137
VIF max	1.223	5.302	5.489

Robust t-statistics in parentheses

*** *p* < 0.01, ** *p* < 0.05, * *p* < 0.1

The variable *MarketCap* is also significant and negative. Apart from Amir et al. (2018), the extant literature repeatedly documented more negative market reactions when data breaches afflicted smaller companies. In many cases, these studies use dummy variables to account for sector-specific reactions (Cavusoglu et al. 2004; Gatzlaff and McCullough 2010; Malhotra and Kubowicz Malhotra 2010). This can be viewed as an indication that while investors react more sensitively in cases involving smaller companies, it is different in the healthcare sector where the costs per stolen record are highest.

*RoE* is also significant and positive. We interpret this result as an indication that the challenges arising from a data breach are viewed differently, depending on the management’s past performance. This finding supports hypothesis 4a. The second financial variable, *IntAssets*, is also highly significant and positive. Firms in the sample primarily reported 3 types of intangible assets (apart from goodwill): hospital agreements, customer relations and patented technology. The variable assesses a company’s reliance on intangible assets to generate sales. This finding indicates that companies whose sales are highly dependent on intangible assets experience less negative reactions from investors. This finding supports hypothesis 4b.

The remaining variables account for the different business activities within the sample. Companies falling under the category *ChemicalsAndMeasuring* experience significant and negative reactions from investors following a data breach. This result stands out as other variables capturing other business activities are not significant, even as they add to the overall explanatory power of the model. From an investor perspective, the fallout from data breaches for companies operating in area of chemicals, measurement and controlling instruments might extend beyond the typical out-of-pocket expenses typically associated with data breaches as these companies more likely own valuable patents. A data breach could therefore put on-going research in jeopardy. Overall, the analysis of industry sectors within healthcare is not conclusive. Still, the findings indicate varying levels of investor reaction, which underlines the varying effects data breaches can have. These findings do not suffice to support hypothesis 2.

The variable *SSN* is negative but insignificant. It is worth noting that of 185 events in the sample, 65 do not have a web description. In those cases, it was not possible to evaluate whether SSN was accessed. This might explain why the evidence does not support hypothesis 3.

## Discussion

This study analyzed stock market reactions following the announcement of data breaches in the healthcare sector. While the analysis of the impact of unauthorized access to SSNs is inconclusive, there are other key findings that merit discussion:

The multivariate regression analysis shows how investor reactions depend on a few key variables: severity of the data breach, past managerial performance (return on equity), sales in relation to intangible assets, size (market capitalization), and business model (sub-industry variables). This evidence underlines the complexity and the great variety within the healthcare sector and why industry-specific analysis is so crucial. For an investor, the key question is whether a data breach will impact a company’s ability to deliver shareholder returns. The presented model suggests that if the management of a company has a good track record, i.e., high return on equity, investors seem less likely to consider future returns at risk. Similarly, if companies can guarantee a large part of their future sales through agreements with hospitals or similar, thereby locking in their customers, investors seem to assume that the negative impact of a data breach is similarly limited.

The main finding, that the average data breach does not result in losses in firm value, differs from most cross-industry studies. While this may partially be due to the study’s focus on healthcare companies, the regulatory environment and the enforced publication requirements seem also important to reflect. The research report from the U.S. Department of Health & Human Services is, due to the mandatory disclosure requirements, the most comprehensive sector-specific report for data breaches available. As a result, the sample can be considered representative of the general population (i.e. data breaches in the healthcare sector affecting more than 500 individuals). Combined with the study’s findings, this underlines the value of such comprehensive databases for research purposes. From a policy perspective reporting, minor data breaches might be a detractor. The sheer number of reported cases creates a level of noise, where serious deficiencies might be easily overlooked. If the goal is to strengthen privacy and security provisions, as laid out in the HITECH Act, the question arises whether the better long-term play might be to highlight fewer but more severe cases. Similarly, one needs to account for compliance costs. Previous studies have highlighted the difficulties smaller organizations face when trying to comply with HIPAA (Chen and Benusa 2017). However, the model shows that bigger firms experience more negative investor reactions. Since the loss in firm value reflects short- and long-term costs, we can assume that investors estimate the compliance costs following a data breach to be substantial.

One limitation of this research lies in its reliance on short-term stock market reaction following the announcement of a data breach. Key findings such as the impact of a high return on equity or a high degree of intangible assets in proportion to sales reflect investors’ attempts at estimating the severity and the likelihood of long-term implications resulting from a data breach. Consequently, an analysis of litigations, legal fees and other types of incurred costs might yield insightful results. In this context, it is also important to reflect changes in the regulatory environment in a broader sense. The HITECH act and disclosure requirements have been the focus of this study. However, other pieces of legislation, such as the Affordable Care Act (ACA), have also reshaped large parts of the healthcare sector. Beyond the United States, the General Data Protection Regulation (GDPR) is impacting multinational companies, as evidenced by numerous references in annual risk disclosures. Another limitation of this study is therefore related to changes in investor perceptions because of major regulatory changes.

## Data Availability

The datasets generated during and/or analyzed during the current study are available from the corresponding author on reasonable request.

## References

[CR1] Amir E, Levi S, Livne T (2018). Do Firms Underreport Information on Cyber-Attacks? Evidence from Capital Markets. Rev Account Stud.

[CR2] Andoh-Baidoo FK, Amoako-Gyampah K, Osei-Bryson KM (2010). How Internet Security Breaches Harm Market Value. IEEE Secur Priv Mag.

[CR3] Angst CM, Block ES, D’Arcy J, Kelley K (2017). When Do It Security Investments Matter? Accounting for the Influence of Institutional Factors in the Context of Healthcare Data Breaches. MIS Q.

[CR4] Avery A, Ranganathan C (2016) Financial performance impacts of information security breaches. Workshop on Information Security and Privacy (WISP), Dublin, Ireland, pp 1–16

[CR5] Bolster P, Pantalone CH, Trahan EA (2010). Security breaches and firm value. J Bus Val Econ Loss Anal.

[CR6] Campbell K, Gordon L, Loeb M, Zhou L (2003). The economic cost of publicly announced information security breaches: Empirical evidence from the stock market. J Comput Secur.

[CR7] Cavusoglu H, Mishra B, Raghunathan S (2004). The Effect of internet security breach announcements on market value: Capital market reactions for breached firms and internet security developers. Int J Electron Commer.

[CR8] Centers for Disease Control and Prevention (2018) Health insurance portability and accountability act of 1996 (HIPAA). Retrieved 2021–04–25, 2021, from https://www.cdc.gov/phlp/publications/topic/hipaa.html#:~:text=The%20Health%20Insurance%20Portability%20and,the%20patient%27s%20consent%20or%20knowledge

[CR9] Centers for Medicare and Medicaid Services (2020) national health expenditures 2019 highlights. Retrieved 2021–04–25, 2021, from https://www.cms.gov/files/document/highlights.pdf

[CR10] Chen JQ, Benusa A (2017). HIPAA security compliance challenges: the case for small healthcare providers. International Journal of Healthcare Management.

[CR11] Dehning B, Richardson VJ, Zmud RW (2003). The value relevance of announcements of transformational information technology investments. MIS Q.

[CR12] Department of Health and Human Services Office for Civil Rights (2021) Breach Portal Research Report. (Retrieved 2021–02–18)

[CR13] Gatzlaff KM, McCullough KA (2010). The effect of data breaches on shareholder wealth. Risk Management and Insurance Review.

[CR14] Goel S, Shawky HA (2014). The impact of federal and state notification laws on security breach announcements. Commun Assoc Inf Syst.

[CR15] Gordon LA, Loeb MP, Zhou L (2011). The impact of information security breaches: has there been a downward shift in costs?. J Comput Secur.

[CR16] Heath M, Porter TH, Silvera G (2022). Hospital characteristics associated with HIPAA breaches. International Journal of Healthcare Management.

[CR17] Hinz O, Nofer M, Schiereck D, Trillig J (2015). the influence of data theft on the share prices and systematic risk of consumer electronics companies. Inf Manag.

[CR18] Kamiya S, Kang J-K, Kim J, Milidonis A, Stulz R (2018) What Is the impact of successful cyberattacks on target firms? National Bureau of economic research working paper series (No. 24409)

[CR19] Kannan K, Rees J, Sridhar S (2007). Market reactions to information security breach announcements: an empirical analysis. Int J Electron Commer.

[CR20] Li H, Yoo S, Kettinger W (2019) The changing tides of investments and strategies and their impacts on security breaches. ICIS 2019 Proceedings. 33. https://aisel.aisnet.org/icis2019/cyber_security_privacy_ethics_IS/cyber_security_privacy/33

[CR21] MacKinlay AC (1997). Event studies in economics and finance. J Econ Lit.

[CR22] Malhotra A, KubowiczMalhotra C (2010). Evaluating customer information breaches as service failures: an event study approach. J Serv Res.

[CR23] McShane M, Nguyen T (2020). Time-varying effects of cyberattacks on firm value. Geneva Pap Risk Insur Issues Pract.

[CR24] Michel A, Oded J, Shaked I (2019). Do Security Breaches Matter? The Shareholder Puzzle. Eur Financ Manag.

[CR25] Modi SB, Wiles MA, Mishra S (2015). Shareholder value implications of service failures in triads: the case of customer information security breaches. J Oper Manag.

[CR26] Pirounias S, Mermigas D, Patsakis C (2014). The relation between information security events and firm market value, empirical evidence on recent disclosures: an extension of the Glz study. J Inf Secur Appl.

[CR27] Ponemon Institute (2016). Sixth annual benchmark study on privacy & security of healthcare data.

[CR28] Ponemon Institute (2017). 2017 Cost of Data Breach Study.

[CR29] S&P Dow Jones Indices (2021) Investment theme sectors. from https://www.spglobal.com/spdji/en/landing/investment-themes/sectors/ (Retrieved 2021–08–15)

[CR30] Song Z, Wang G, Fan W (2017) Firm actions toward data breach incidents and firm equity value: an empirical study. 10.24251/HICSS.2017.602

[CR31] Subramani M, Walden E (2001). The impact of e-commerce announcements on the market value of firms. Inf Syst Res.

[CR32] Tanriverdi H, Kwon J, Im G (2020) Data breaches in multihospital systems: antecedents and mitigation mechanisms. ICIS 2020 Proceedings. 10. https://aisel.aisnet.org/icis2020/cyber_security_privacy/cyber_security_privacy/10

[CR33] U.S. Department of Health & Human Services (2017) Hitech act enforcement interim final rule. from https://www.hhs.gov/hipaa/for-professionals/special-topics/hitech-act-enforcement-interim-final-rule/index.html#:~:text=The%20Health%20Information%20Technology%20for,use%20of%20health%20information%20technology. (Retrieved 2021–04–25)

[CR34] Yayla AA, Hu Q (2011). The impact of information security events on the stock value of firms: the effect of contingency factors. J Inf Technol.

[CR35] Zafar H, Ko M, Osei-Bryson K-M (2009) Intra-industry effects of information security breaches on firm performance. AMCIS 2009 Proceedings. 590. https://aisel.aisnet.org/amcis2009/590

